# Fixation of Takeuchi Type II/III lateral hinge fractures provides favourable stability of a medial open wedge high tibial osteotomy—A biomechanical study

**DOI:** 10.1002/ksa.12560

**Published:** 2024-12-15

**Authors:** Christian Peez, Adrian Deichsel, Ivan Zderic, R. Geoff Richards, Ludmil Drenchev, Hristo K. Skulev, Boyko Gueorguiev, Michael J. Raschke, Christoph Kittl, Elmar Herbst

**Affiliations:** ^1^ AO Research Institute Davos Davos Switzerland; ^2^ Department of Trauma, Hand and Reconstructive Surgery University Hospital Münster Münster Germany; ^3^ Bulgarian Academy of Sciences, Institute of Metal Science “Acad. A. Balevski” Sofia Bulgaria

**Keywords:** lateral hinge fractures, medial open wedge high tibial osteotomy, motion tracking, plate, screw, Takeuchi classification, torsional instability

## Abstract

**Purpose:**

To investigate the biomechanical consequences of osteosynthesis of lateral hinge fractures (LHFs) in medial open wedge high tibial osteotomy (MOWHTO).

**Methods:**

Sixteen fresh‐frozen human cadaveric proximal tibiae underwent MOWHTO fixed with an ipsilateral locking compression plate. The specimens were assigned to two clusters simulating LHFs according to the Takeuchi classification: (1) Type II fracture; and (2) Type III fracture. The following conditions were serially tested: (1) intact hinge; (2) fractured hinge; (3) screw fixation of the LHF; (4) staple fixation of the LHF; and (5) locking T‐plate fixation of the LHF. Each specimen was subjected to 10 cycles of axial compression load (720 N; 36 N/s), and internal and external rotational loads (10 N m; 1 N m/s), while capturing the interfragmentary movements via motion tracking.

**Results:**

In Takeuchi Type II fractures, osteosynthesis of the fractured hinge with staples or a plate significantly reduced fracture site displacement (*p* < 0.05) and significantly increased construct stiffness (*p* < 0.05) under axial and torsional loading, while only the plate restored intact torsional displacement (n.s.). For Takeuchi Type III fractures, both screw and plate fixation significantly reduced fracture site displacement (*p* < 0.05) and significantly increased construct stiffness (*p* < 0.05) under axial and torsional loading. Both techniques restored torsional stiffness in each rotational direction and torsional displacement in internal rotation (n.s.).

**Conclusion:**

Additional plate fixation of Takeuchi Type II fractures was the construct with the highest stiffness, restoring the axial and torsional stability to a MOWHTO with an intact hinge. Screw and plate fixation of Takeuchi Type III fractures provided equivalent stability and restored the torsional and axial stability of the MOWHTO. In case of a Takeuchi Type II or III fracture, surgeons should consider additional plate or screw osteosynthesis of the fractured hinge to best restore the stability of the MOWHTO, which may potentially reduce the risk of loss of correction and impaired bone healing.

**Level of Evidence:**

There is no level of evidence as this study was an experimental laboratory study.

**Abbreviations**
BMD: bone mineral density
CT: computed tomography
ER: external rotation
IR: internal rotation
LHF: lateral hinge fracture
MOWHTO: medial open wedge high tibial osteotomy
PMMA: polymethylmethacrylate
SD: standard deviation

## INTRODUCTION

Valgus‐producing medial open wedge high tibial osteotomy (MOWHTO) is a well‐established procedure for treatment of varus deformity with mild to moderate compartment osteoarthritis [7, 17], chronic posterolateral knee instability [3], medial meniscal deficiency [33] and in combination with cartilage repair procedures [1]. With selective indication, accurate correction of lower limb alignment, and stable fixation allowing for early post‐operative rehabilitation, MOWHTO has demonstrated excellent clinical, functional and radiographic outcomes with survival rates of approximately 80% at 10 years [6, 15, 19]. However, perioperative complication rates remain high, with lateral hinge fractures (LHFs) and malunion of the osteotomy gap being the major clinical concerns [24].

These fractures at the hinge of the osteotomy site may occur with an incidence of up to 50% [20, 21, 22] and are present with three different fracture types: Type I, extension into the proximal tibiofibular joint; Type II, extension distal to the proximal tibiofibular joint; and Type III, extension into the lateral tibial plateau [26, 32]. In particular, Takeuchi fracture Types II and III are considered unstable [26, 32] and increase the risk of delayed union or non‐union of the osteotomy gap, resulting in fatigue‐induced hardware failure, loss of correction, and chronic post‐operative pain [8, 12, 18, 30]. In this context, the altered biomechanical environment with reduced axial and torsional stability of the bone‐implant construct has been discussed as a critical factor for impaired bone healing [4, 16]. As a result, several strategies have been proposed to prevent these fractures, including the use of protective hinge wires [14] and positioning of the lateral hinge within the safe zone at the level of the proximal tibiofibular joint [25]. Despite these efforts, a certain rate of LHFs remains [14, 25].

Therefore, the aim of this study was to examine the biomechanical consequences of osteosynthesis of unstable Type II and III LHFs related to MOWHTO. It was hypothesized that (1) additional fixation of Takeuchi Type II and III fractures would restore the MOWHTO stability and (2) the stabilizing effect of the fixation technique depends on the Takeuchi fracture type.

## MATERIALS AND METHODS

### Specimens and preparation

Institutional Review Board approval was obtained from the AO Research Institute Davos to conduct this biomechanical study (PP2115, 6 February 2018). All donors gave informed consent to the use of their corpses in medical science during their lifetime, so that the specimens were dissected and biomechanically tested in accordance with the relevant guidelines and regulations.

Sixteen fresh‐frozen (−20°C) non‐paired human cadaveric knees from 4 female and 12 male donors aged 72.2 ± 7.0 years (mean ± standard deviation, SD) (range 59–82 years) were obtained from an international tissue bank (Science Care). Specimens with previous surgery, previous tibial fracture, high‐grade osteoarthritis (Kellgren–Lawrence Grade III and IV), or evidence of ligament damage were excluded. During dissection, specimens were examined to ensure the integrity of the proximal tibia and proximal tibiofibular joint, and absence of advanced osteoarthritis.

Bone mineral density (BMD) within the trabecular region of the proximal tibial metaphysis was assessed in each specimen using computed tomography (CT) scanning (Revolution EVO, General Electric Healthcare). A phantom (BDC‐6, QRM GmbH) was subsequently analyzed using an image processing software (Amira, v.6.0, Thermo Fisher Scientific) with segmentation between 150 and 450 mgHA/cm^3^.

Based on BMD, the knees were assigned to two clusters consisting of eight specimens each (*n* = 8), with homogenous BMD distribution between the two clusters (n.s.). Within each cluster, a different type of LHF was simulated according to the Takeuchi classification [32]: (1) Type II fractures extending distal to the proximal tibiofibular joint and (2) Type III fractures extending into the lateral tibial plateau.

Prior to preparation and biomechanical testing, the knees were thawed at room temperature for 24 h. The tibia and fibula were then cut 250 mm distal to the knee joint line, and the knee joints were disarticulated to harvest the proximal lower leg. The entire soft tissue was dissected while preserving the interosseous membrane and articular capsule of the proximal tibiofibular joint. Once the fibula was secured to the tibia in its anatomical position with a 3.5 mm tri‐cortical position screw, the specimens were wrapped in phosphate‐buffered saline‐soaked tissue paper to prevent tissue dehydration during specimen preparation.

**Figure 1:**
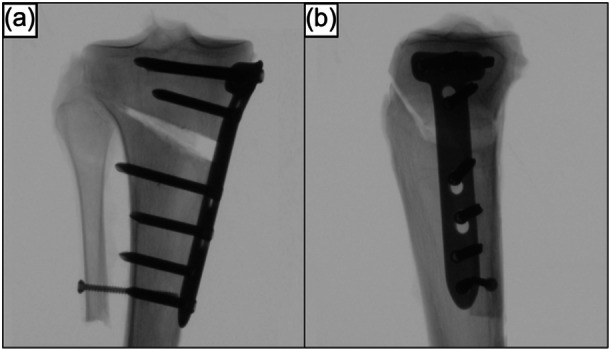
Anteroposterior (a) and mediolateral (b) radiographs of a right proximal tibia after a biplanar, medial open wedge high tibial osteotomy fixed with a locking compression plate. Care was taken to preserve an intact lateral hinge.

Each specimen underwent a biplanar MOWHTO using the technique described by Palmer et al. [27] with subtle modifications. The osteotomy plane was marked with two parallel 2.4 mm Kirschner (K‐) wires with the tips placed lateral to the medial margin of the proximal tibiofemoral joint, marking the hinge position in the safe zone [25]. For the biplanar cut, an ascending anterior osteotomy was performed behind the tibial tuberosity at an angle of 100° to the planned correction level, followed by an axial osteotomy along the K‐wires using an oscillating saw. Consistently with a previous study [4], the osteotomy gap was opened to a height of 10 mm, while preserving an intact lateral hinge of 10 mm width. Then, the MOWHTO was fixed using a medial locking compression plate (LCP) system (4.5/5.0 mm LCP; TomoFix medial proximal tibia; Johnson & Johnson MedTech). Four unicortical locking screws were placed in the metaphyseal segment, while the diaphyseal segment was fixed with four bicortical locking screws (Figure 1).

Upon completion of the surgical procedures, the distal 6 cm of the tibial shafts were embedded in a polymethylmethacrylate (PMMA, Suter Kunststoffe AG) socket. Subsequently, the tibial plateau was embedded in another PMMA socket, leaving lateral 50% of the lateral tibial plateau and the proximal tibiofibular joint free of PMMA. Finally, retro‐reflective marker sets were attached to the tibial shaft, the lateral hinge site, and the medial proximal tibial metaphysis for motion tracking.

### Testing conditions and workflow

**Figure 2:**
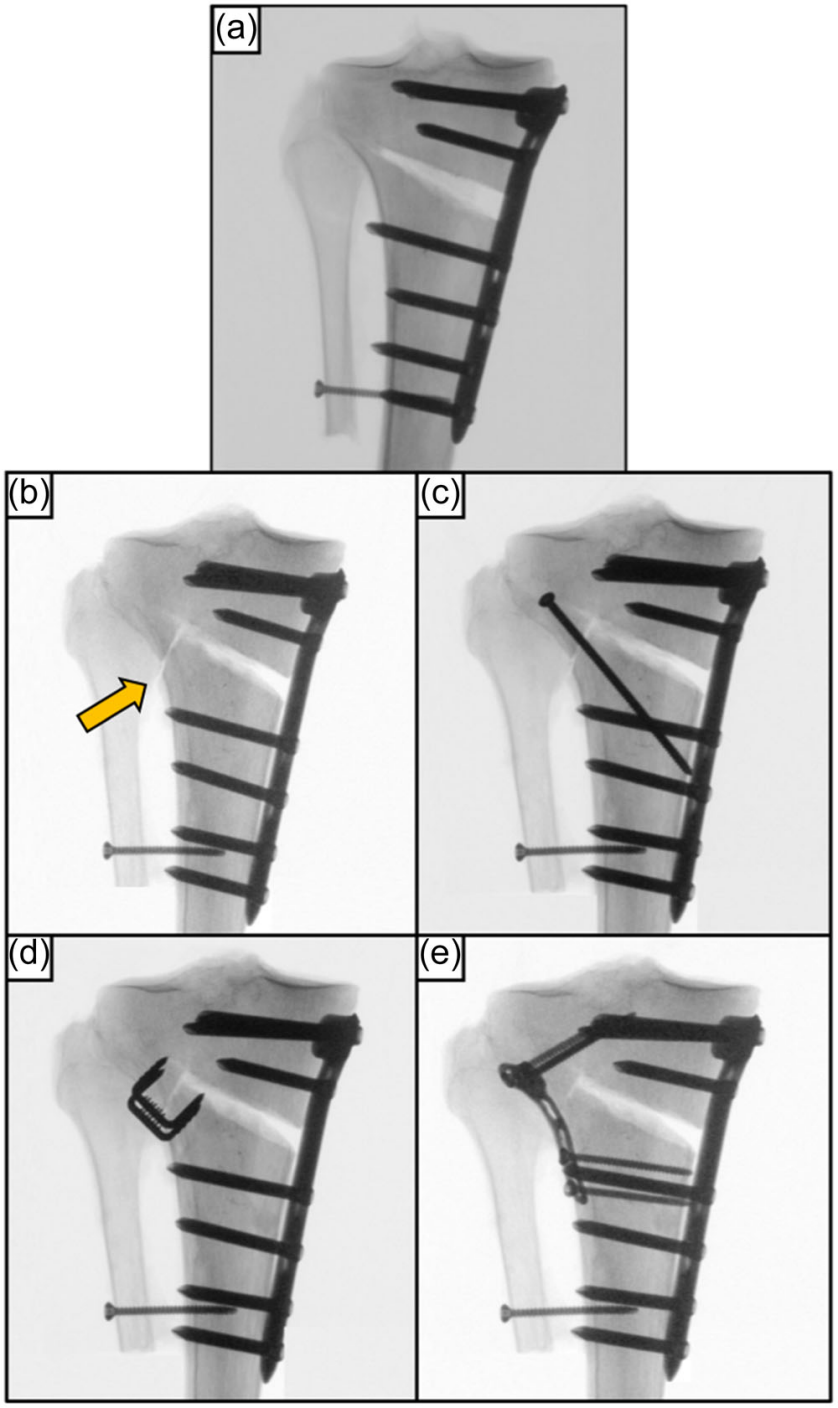
Anteroposterior radiographs of a right proximal tibia after a biplanar, medial open wedge high tibial osteotomy fixed with an ipsilateral locking compression plate system. (a) Preserved lateral hinge (intact). (b) Construct with a lateral hinge fracture (LHF) extended distal to the proximal tibiofibular joint (Takeuchi Type II fracture; orange arrow). (c) Additional lag screw fixation of the LHF (screw). (d) Additional staple fixation of the LHF (staples). (e) Additional T‐plate fixation of the LHF (plate).

The following conditions of the specimens assigned to the Takeuchi Type II fracture cluster were serially tested: (1) MOWHTO with preserved hinge (intact); (2) MOWHTO with Takeuchi Type II LHF (fracture); (3) MOWHTO with additional screw fixation of the LHF (screw); (4) MOWHTO with additional staple fixation of the LHF (staples); and (5) MOWHTO with additional locking T‐plate fixation of the LHF (plate) (Figure 2).

**Figure 3:**
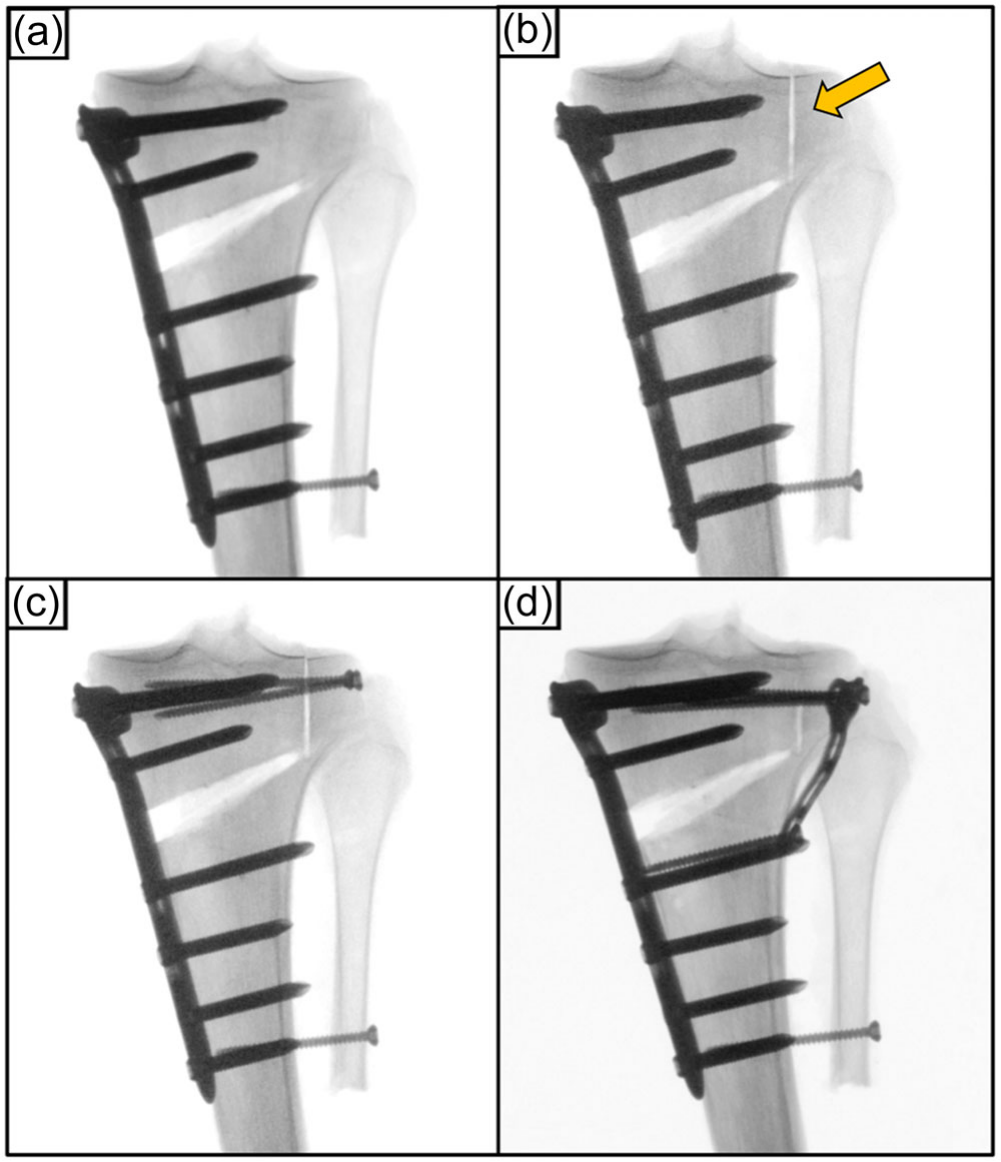
Anteroposterior radiographs of a left proximal tibia after a biplanar, medial open wedge high tibial osteotomy fixed with an ipsilateral locking compression plate system. (a) Preserved lateral hinge (intact). (b) Construct with a lateral hinge fracture (LHF) extended into the lateral tibial plateau (Takeuchi type III fracture; orange arrow). (c) Additional lag screw fixation of the LHF (screw). (d) Additional T‐plate fixation of the LHF (plate).

The specimens assigned to the Takeuchi Type III fracture cluster were serially tested in the following conditions: (1) MOWHTO with preserved hinge (intact); (2) MOWHTO with Takeuchi Type III LHF (fracture); (3) MOWHTO with additional screw fixation of the LHF (screw); and (4) MOWHTO with additional locking T‐plate fixation of the LHF (plate) (Figure 3).

The fracture was simulated according to the Takeuchi classification [32]. Based on the cluster assignment, the lateral hinge was either osteotomized with distal extension to the proximal tibiofibular joint (Type II fracture) (Figure 2b) or with proximal extension into the lateral tibial plateau (Type III fracture) (Figure 3b) using an oscillating saw.

Screw instrumentation of the LHF was performed according to the author's clinical practice to provide a stable fixation that best neutralizes axial compression and torsional loading [10, 12]. The Takeuchi Type II LHFs were fixed with a 3.5 mm cortical lag screw of 60 mm length (Johnson & Johnson MedTech) inserted from Gerdy's tubercle, targeting the posteromedial cortex and crossing the fractured hinge perpendicularly (Figure 2c). For Takeuchi Type III fractures, two 3.5 mm screws of 50 mm length each were inserted from lateral to medial and parallel to the tibial plateau (Figure 3c), providing a two‐point fixation of the fractured hinge.

Staple fixation in the Takeuchi Type II fracture was assured with two stainless steel staples of 14 mm width and 15 mm length (Richard Fixation Staple, Smith & Nephew). The bone staples were inserted perpendicular to the anterolateral cortex of the proximal tibia (Figure 2d). The osteotomy gap was inspected after osteosynthesis to exclude protrusion of the staple tines into the osteotomy gap.

LHF plate fixation used a 3.5 mm locking T‐plate (3.5 mm T‐LCP; Johnson & Johnson MedTech). In Takeuchi Type II fractures the proximal cortical screws were directed toward the intercondylar spines (Figure 2e), whereas in the Takeuchi Type III fractures the screws were inserted parallel to the tibial plateau (Figure 3d). Distally, the T‐plate was fixed with two cortical screws.

### Biomechanical testing

**Figure 4:**
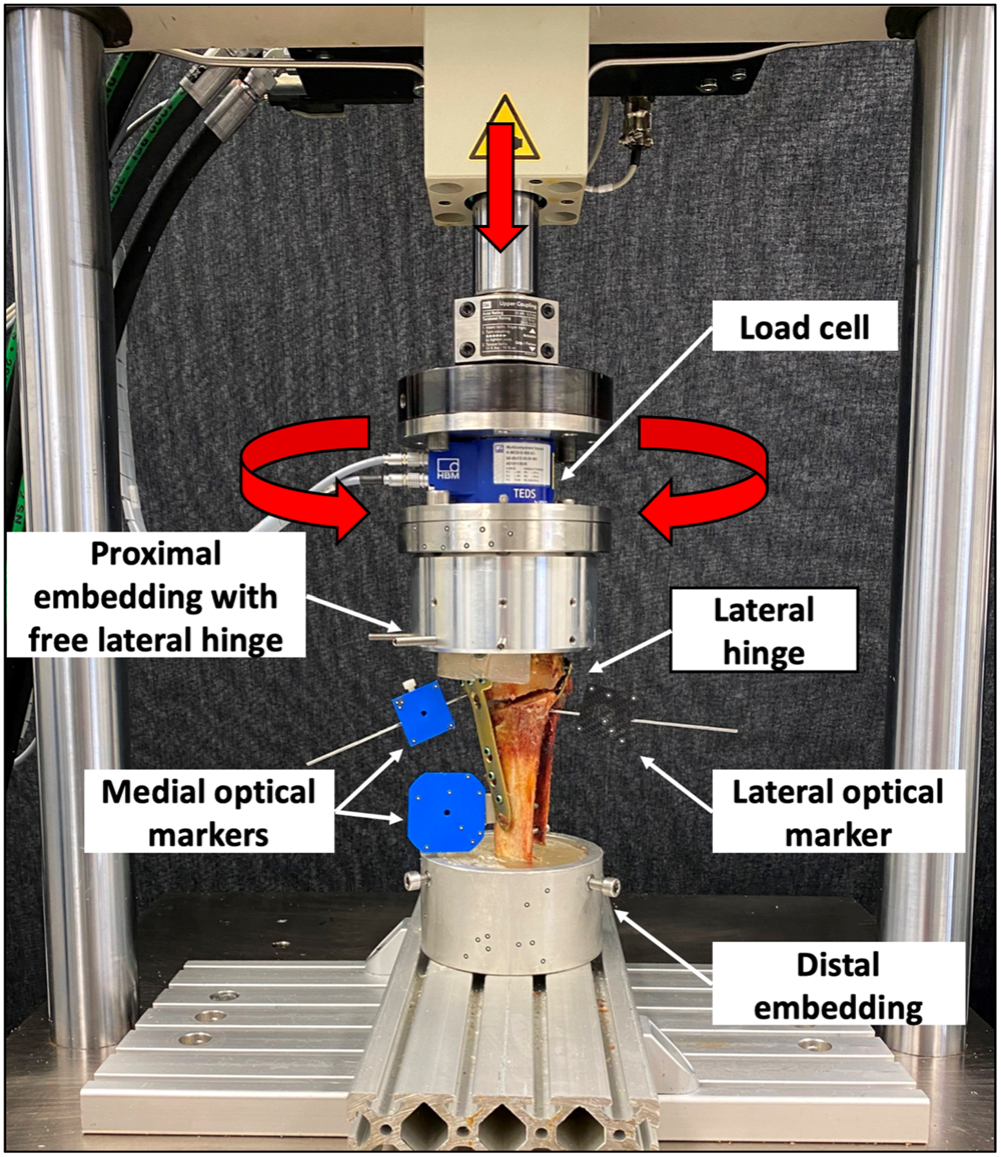
Setup with a specimen mounted for biomechanical testing. The vertical arrow denotes loading direction for axial loading, the semi‐circular arrows denote the bidirectional torsional loading directions.

Biomechanical testing was performed using a servo‐hydraulic materials testing machine (Bionix 858.20, MTS Systems Corp.) equipped with a 5 kN load cell (HBM). Each specimen was tested in an upright standing position, with the distal tibial PMMA socket rigidly mounted to the machine base plate. Axial compression and torsional loading along the machine axis were applied via a custom‐made flange connected to the proximal tibial PMMA socket, allowing a homogenous load transfer to the metaphyseal segment of the proximal tibia without restricting the mobility of the lateral hinge and proximal tibiofibular joint (Figure 4).

Starting with a preserved hinge condition (intact), each specimen was axially loaded with 10 cycles of a non‐destructive quasi‐static ramp (720 N, 36 N/s), followed by torsional loading with 10 cycles of a non‐destructive quasi‐static ramp (10 Nm, 1 Nm/s) in internal and external rotation (IR/ER) as previously described [28]. Once the measurements were completed in the intact test condition, the lateral hinge site was osteotomized to create LHFs according to the specimen's treatment assignment. Then, the biomechanical testing and measurements were repeated for the fractured lateral hinge status and the different techniques of hinge fixation as described above. For each test, the specimens were wrapped in phosphate‐buffered saline‐soaked tissue paper to prevent tissue dehydration.

### Data acquisition and evaluation

Axial displacement, axial load, torsional angle and torque were continuously recorded by the test system controllers at 128 Hz during each test. Based on these data, force–displacement and torque–angle curves were generated to calculate the axial and torsional construct stiffness, defined as the slope of the last quasi‐static ramp.

A stereographic optical motion tracking system using contactless full‐field deformation technology (Aramis SRX, Carl Zeiss GOM Metrology GmbH) continuously captured the coordinates of the attached optical markers in all six degrees of freedom, operating at a resolution of 12 MP and a maximum acceptance error of 0.004 mm [29]. Based on this, interfragmentary movements were recorded for each of the 10 test cycles and analyzed as the mean value among the cycles in loaded condition. Specifically, fracture site displacement along the tibial shaft axis—defined as axial displacement—and perpendicular to it in anteroposterior direction—defined as torsional displacement—was calculated based on the displacement of the lateral border of the hinge osteotomy. In addition, axial displacement of the medial osteotomy gap was captured at the most medial gap margin. For this purpose, the location of the lateral hinge border and the most medial osteotomy gap margin were registered with a touch probe prior to test start and virtually assigned in a rigid‐body constellation (1) once to the proximal and distal fragment of the LHF and (2) once to the proximal and distal fragment of the medial osteotomy gap. Based on this, the relative displacement between these virtually registered margins was calculated along the tibial shaft axis and perpendicular to it in anteroposterior direction. Furthermore, interfragmentary rotation around the longitudinal axis of the tibial shaft—defined as hinge rotation—was evaluated.

### Statistical analysis

Statistical analysis was performed using Prism (Version 9, GraphPad Software). Descriptive data are presented as mean values ± SD. Normality of data distribution within each fixation technique was tested and proved using the Shapiro–Wilk test, followed by an Independent‐Samples *T*‐test to confirm the appropriate randomization of the specimens based on BMD. Significant differences between the tested conditions regarding construct stiffness, axial displacement, torsional displacement, and hinge rotation were identified using repeated measures of two‐way ANOVA followed by Dunnett post hoc test for multiple comparisons. The overall level of significance was set at 0.05.

An a‐priori power analysis was performed using G*Power‐2 software (University Düsseldorf) [9]. Based on mean values and standard deviations from a previous study evaluating the displacement of hinge fractures after varisation distal femoral osteotomies [28], it was assumed that a sample size of 6 would allow identification of changes in displacement of 0.3 mm with an SD of 0.2 mm (effect size/Cohen's *d* = 1.5) with 80% power, at a level of significance of 0.05.

## RESULTS

### Takeuchi Type II fracture

**Figure 5:**
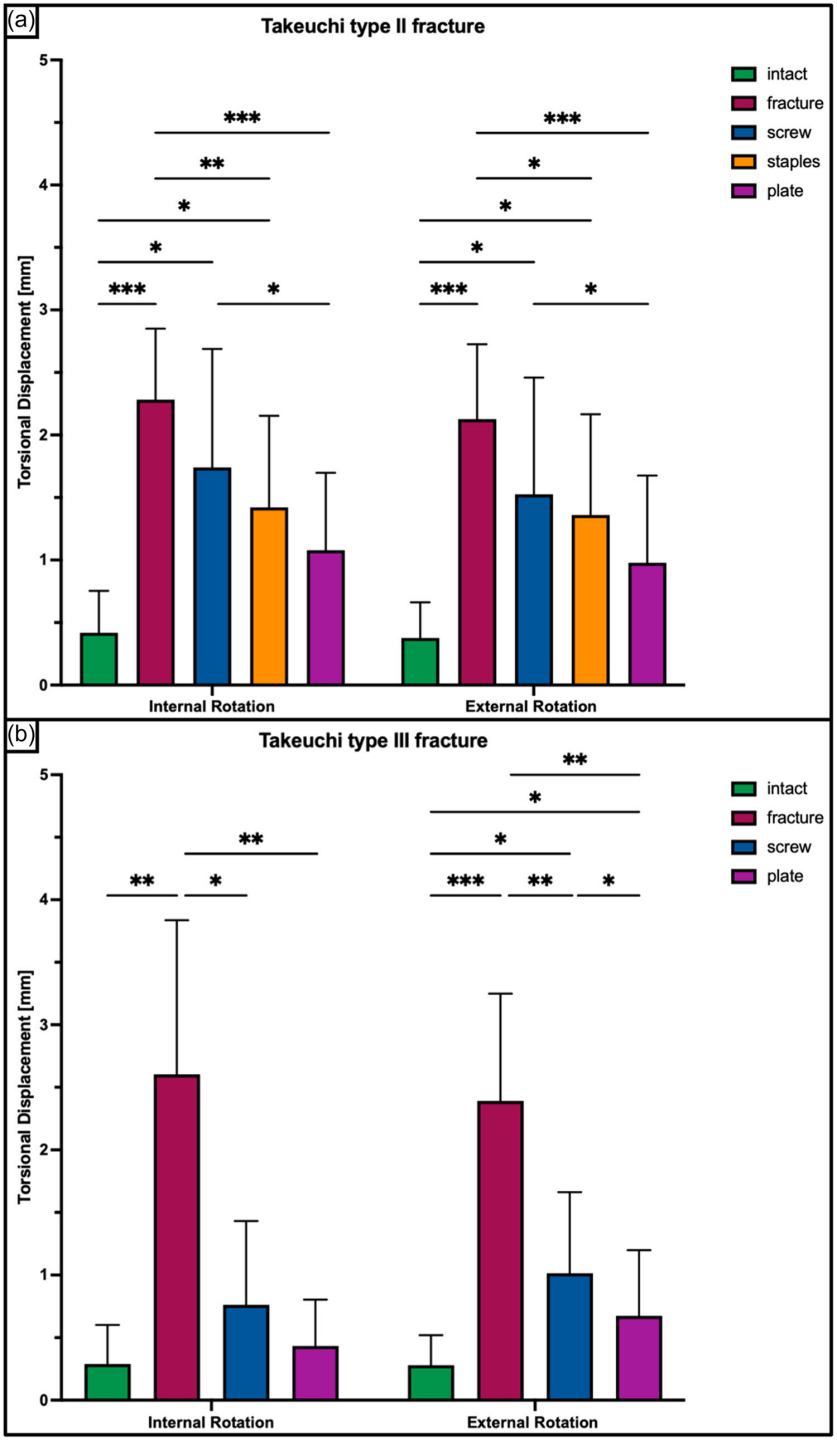
Torsional displacement in internal and external rotation shown for each tested condition separately in terms of mean value ± standard deviation. (a) Takeuchi Type II fracture. (b) Takeuchi Type III fracture. The asterisks (*) indicate significant differences with *p* < 0.05. mm, millimetre.

**Figure 6:**
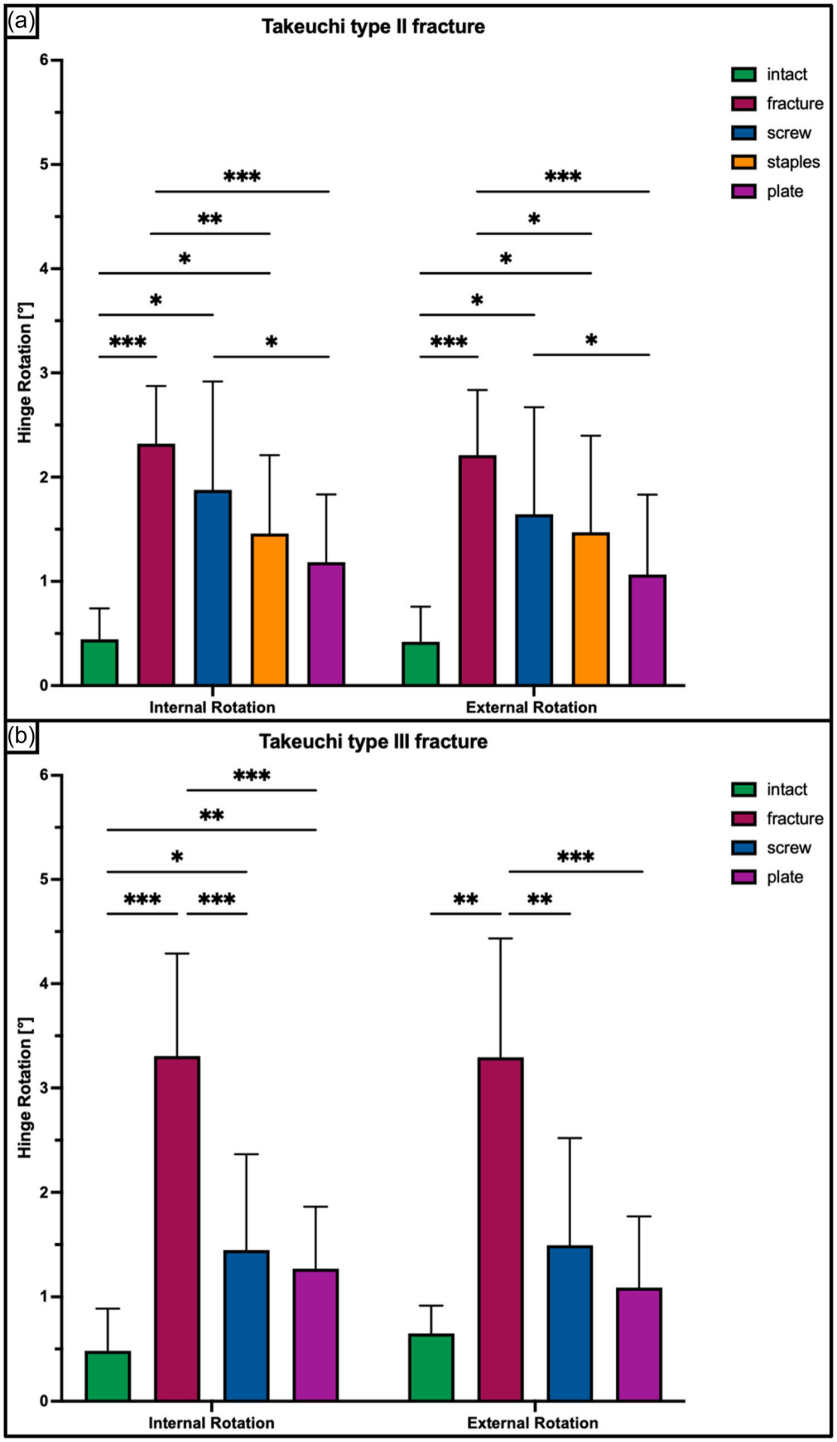
Hinge rotation in internal and external rotation shown for each tested condition separately in terms of mean value ± standard deviation. (a) Takeuchi Type II fracture. (b) Takeuchi Type III fracture. The asterisks (*) indicate significant differences with *p* < 0.05. °, degree.

**Figure 7:**
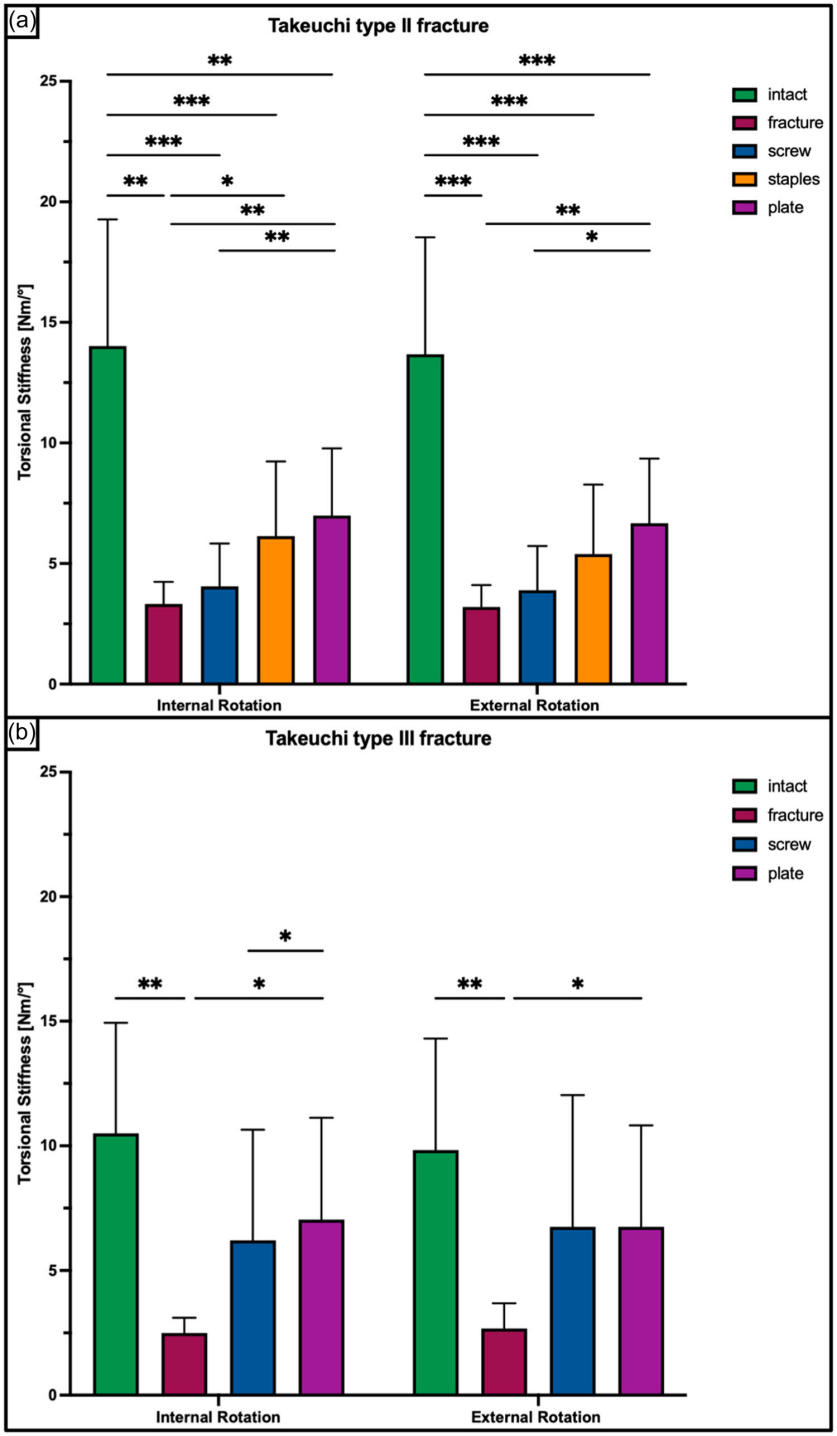
Torsional stiffness in internal and external rotation shown for each tested condition separately in terms of mean value ± standard deviation. (a) Takeuchi Type II fracture. (b) Takeuchi Type III fracture. The asterisks (*) indicate significant differences with *p* < 0.05. Nm/°, Newtonmetre per degree.

**Figure 8:**
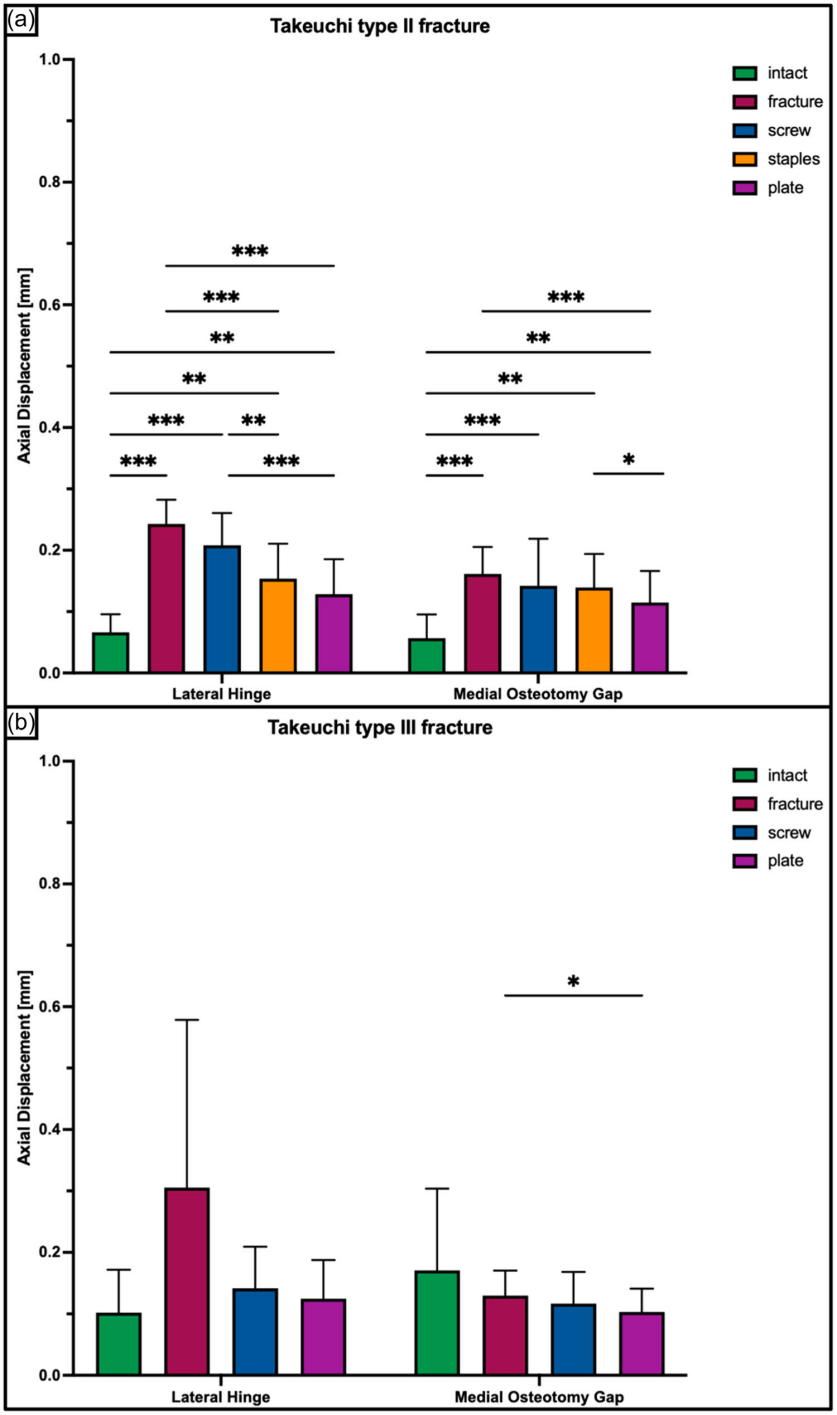
Axial displacement of the lateral hinge and medial osteotomy gap shown for each tested condition separately in terms of mean value ± standard deviation. (a) Takeuchi Type II fracture. (b) Takeuchi Type III fracture. The asterisks (*) indicate significant differences with *p* < 0.05. mm, millimetre.

**Figure 9:**
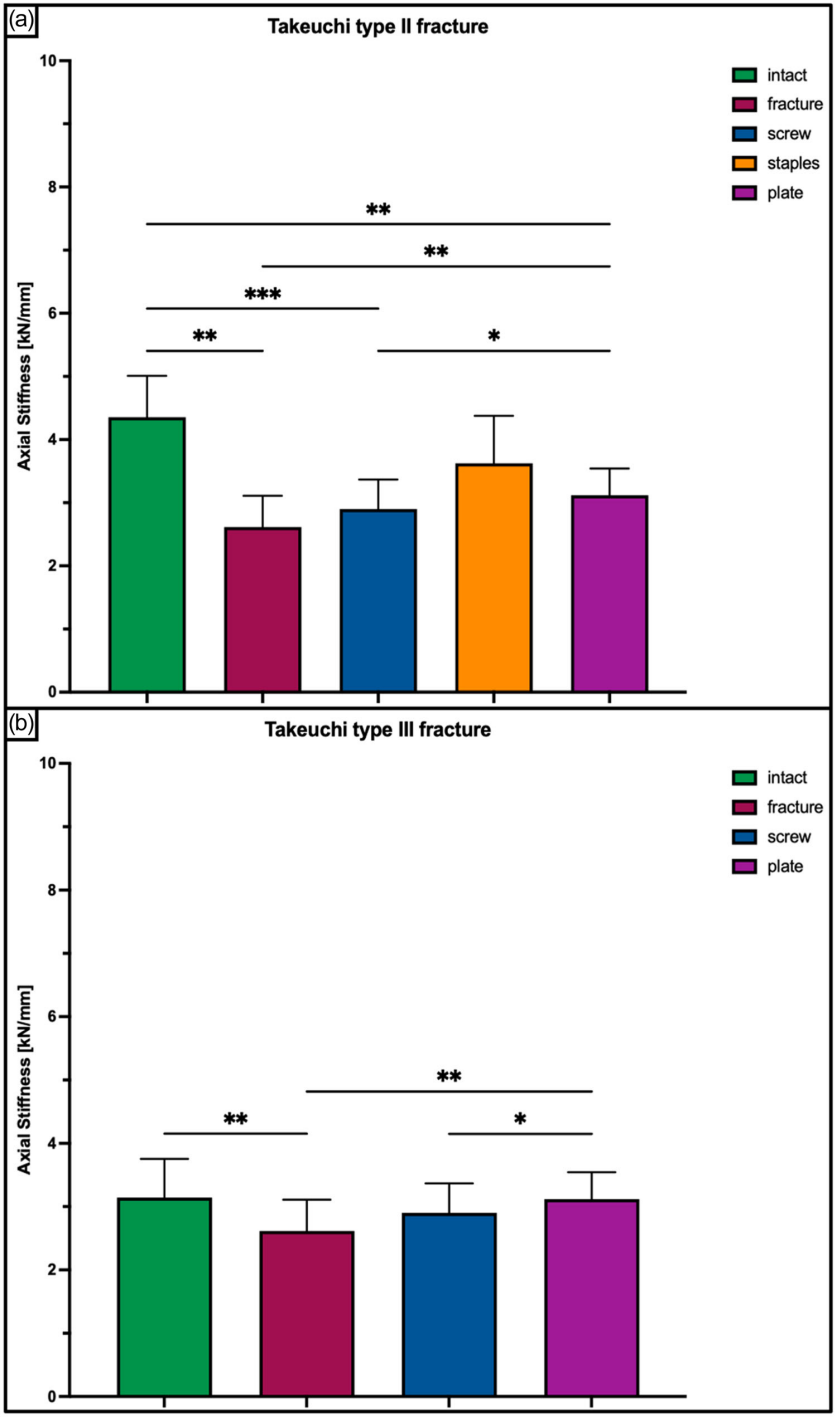
Axial stiffness shown for each tested condition separately in terms of mean value ± standard deviation. (a) Takeuchi Type II fracture. (b) Takeuchi Type III fracture. The asterisks (*) indicate significant differences with *p* < 0.05. kN/mm, kilo Newton per millimetre.

A Takeuchi Type II fracture significantly increased torsional displacement (*p* < 0.05) and hinge rotation (*p* < 0.05), and reduced torsional stiffness (*p* < 0.05) of the bone‐implant construct as compared to an intact hinge under torsional loading. This resulted in 2.3 ± 0.6 mm increased torsional displacement at the hinge site and 2.3 ± 0.5° increased hinge IR, as well as 80% decreased stiffness in each rotational direction (Figures 5a, 6a, and 7a). Under axial loading, a fractured hinge significantly increased axial displacement across the osteotomy gap (*p* < 0.05), resulting in 0.2 mm increased axial displacement at both the lateral hinge site and the medial osteotomy gap (Figure 8a) and 72% reduced axial construct stiffness (Figure 9a).

Osteosynthesis of the fractured hinge with staples or a plate significantly reduced fracture site displacement (*p* < 0.05) and significantly increased construct stiffness (*p* < 0.05) under axial and torsional loading. Neither a screw osteosynthesis nor staples or a plate could restore torsional stiffness to normal (*p* < 0.05). Torsional displacement and hinge rotation were only restored by plate osteosynthesis (n.s). In each rotational direction, the plate performed better compared to the screw and staples, with significantly higher values for torsional stiffness (+56% and +27%; *p* < 0.05) and lower values for torsional displacement (−62% to −33%, *p* < 0.05) (Figures 5a, 6a, and 7a). In contrast to torsional loading, no additional hinge fixation technique could restore intact axial displacement and axial stiffness under axial loading (*p* < 0.05), except for the axial stiffness after staple fixation (n.s.) (Figures 8a and 9a).

### Takeuchi Type III fracture

Under torsional loading, a Takeuchi Type III fracture caused significantly increased torsional displacement (*p* < 0.05) and hinge rotation (*p* < 0.05) of 2.6 ± 1.2 mm and 3.3 ± 0.9°, respectively (Figures 5b and 6b). Complementary, this resulted in a significantly reduced torsional stiffness of 77% in each rotational direction compared to the intact state (*p* < 0.05) (Figure 7b). Considering axial loading, a fractured hinge did not significantly affect axial displacement at both the lateral hinge site and the medial osteotomy gap (n.s), while the axial stiffness was significantly reduced by 21% (*p* < 0.05) (Figures 8b and 9b).

Osteosynthesis of the fractured hinge with screws or a plate significantly reduced fracture site displacement (*p* < 0.05) and significantly increased construct stiffness (*p* < 0.05) under axial and torsional loading. Both fixation techniques of Takeuchi type III fractures could restore the torsional stiffness of the MOWHTO back to normal (n.s.) (Figure 7b). In IR, screw and plate osteosynthesis of the fractured hinge restored torsional displacement and hinge rotation (n.s.). Complementary, both techniques showed an equivalent performance with comparable values for torsional stiffness (±8.8%; n.s.) and torsional displacement (±15.2%; n.s.) in each rotational direction (Figures 5b and 7b). Both additional hinge fixation techniques could restore intact axial stiffness and maintain intact axial displacement (n.s.) (Figures 8b and 9b).

## EFFECT OF TAKEUCHI FRACTURE TYPE

Takeuchi Type II and III LHFs treated with screw or plate osteosynthesis showed comparable biomechanical performance with no significant differences in fracture site displacement and construct stiffness under axial and torsional loading within each fixation technique (n.s.) (Tables 1 and 2).

**Table 1:** Mean ± standard deviation of torsional displacement, hinge rotation, torsional stiffness, axial displacement and axial stiffness for the Takeuchi Type II fracture cluster, together with the *p* values following the statistical comparison of the different tested conditions with the intact lateral hinge state.

	Takeuchi Type II fracture				
Parameters	Intact	Fracture	Screw	Staples	Plate
Torsional displacement IR (mm) (*p* value)	0.4 ± 0.3	2.3 ± 0.6 (<0.05)	1.7 ± 0.9 (<0.05)	1.4 ± 0.7 (<0.05)	1.1 ± 0.6 (n.s.)
Torsional displacement ER (mm) (*p* value)	0.4 ± 0.3	2.1 ± 0.6 (<0.05)	1.5 ± 0.9 (<0.05)	1.4 ± 0.8 (<0.05)	1.0 ± 0.7 (n.s.)
Hinge rotation IR (°) (*p* value)	0.4 ± 0.3	2.3 ± 0.6 (<0.05)	1.9 ± 1.0 (<0.05)	1.5 ± 0.8 (<0.05)	1.2 ± 0.7 (n.s.)
Hinge rotation ER (°) (*p* value)	0.4 ± 0.3	2.2 ± 0.6 (<0.05)	1.6 ± 1.0 (<0.05)	1.5 ± 0.9 (<0.05)	1.1 ± 0.8 (n.s.)
Torsional stiffness IR (N m/°) (*p* value)	14.0 ± 5.3	3.3 ± 0.9 (<0.05)	4.1 ± 1.8 (<0.05)	6.1 ± 3.1 (<0.05)	7.0 ± 2.8 (<0.05)
Torsional stiffness ER (N m/°) (*p* value)	13.7 ± 4.6	3.2 ± 0.9 (<0.05)	3.9 ± 1.8 (<0.05)	5.4 ± 2.9 (<0.05)	6.7 ± 2.7 (<0.05)
Axial displacement lateral hinge (mm) (*p* value)	0.1 ± 0.1	0.2 ± 0.1 (<0.05)	0.2 ± 0.1 (<0.05)	0.2 ± 0.1 (<0.05)	0.1 ± 0.1 (<0.05)
Axial displacement medial osteotomy gap (mm) (*p* value)	0.1 ± 0.1	0.2 ± 0.1 (<0.05)	0.1 ± 0.1 (<0.05)	0.1 ± 0.05 (<0.05)	0.1 ± 0.1 (<0.05)
Axial stiffness (kN/mm) (*p* value)	4.4 ± 0.7	2.6 ± 0.5 (<0.05)	2.9 ± 0.5 (<0.05)	3.6 ± 0.8 (n.s.)	3.1 ± 0.4 (<0.05)

Abbreviations: °, degree; ER, external rotation; IR, internal rotation; kN/mm, kilo Newton per millimetre; mm, millimetre; Nm/°, Newtonmetre per degree.

**Table 2:** Mean ± standard deviation of torsional displacement, hinge rotation, torsional stiffness, axial displacement and axial stiffness for the Takeuchi Type III fracture cluster, together with the *p* values following the statistical comparison of the different tested conditions with the intact lateral hinge state.

	Takeuchi Type III fracture			
Parameters	Intact	Fracture	Screw	Plate
Torsional displacement IR (mm) (*p* value)	0.3 ± 0.3	2.6 ± 1.3 (<0.05)	0.8 ± 0.7 (n.s.)	0.4 ± 0.4 (n.s.)
Torsional displacement ER (mm) (*p* value)	0.3 ± 0.2	2.4 ± 0.9 (<0.05)	1.0 ± 0.7 (<0.05)	0.7 ± 0.5 (<0.05)
Hinge rotation IR (°) (*p* value)	0.5 ± 0.4	3.3 ± 1.0 (<0.05)	1.4 ± 1.0 (<0.05)	1.3 ± 0.6 (<0.05)
Hinge rotation ER (°) (*p* value)	0.7 ± 0.3	3.3 ± 1.1 (<0.05)	1.5 ± 1.0 (n.s.)	1.1 ± 0.7 (n.s.)
Torsional stiffness IR (N m/°) (*p* value)	10.5 ± 4.4	2.5 ± 0.6 (<0.05)	6.2 ± 4.4 (n.s.)	7.0 ± 4.1 (n.s.)
Torsional stiffness ER (N m/°) (*p* value)	9.8 ± 4.5	2.7 ± 1.0 (<0.05)	6.8 ± 5.2 (n.s.)	6.8 ± 4.1 (n.s.)
Axial displacement lateral hinge (mm) (*p* value)	0.1 ± 0.1	0.3 ± 0.3 (n.s.)	0.1 ± 0.1 (n.s.)	0.1 ± 0.1 (n.s.)
Axial displacement medial osteotomy gap (mm) (*p* value)	0.2 ± 0.1	0.1 ± 0.1 (n.s.)	0.1 ± 0.1 (n.s.)	0.1 ± 0.1 (n.s.)
Axial stiffness (kN/mm) (*p* value)	3.1 ± 0.6	2.6 ± 0.5 (<0.05)	2.9 ± 0.5 (n.s.)	3.1 ± 0.4 (n.s.)

Abbreviations: °, degree; ER, external rotation; IR, internal rotation; kN/mm, kilo Newton per millimetre; mm, millimetre; Nm/°, Newtonmetre per degree.

## DISCUSSION

The most important finding of the present study was that Takeuchi Type II and III LHFs caused significantly increased fracture site displacement and decreased bone‐implant construct stiffness in MOWHTO. Additional plate fixation of Takeuchi Type II fractures was associated with higher stiffness and least displacement as compared to a screw or staples osteosynthesis. Even if torsional displacement was restored after plate fixation, rotational stiffness persisted. In contrast, both screw and plate osteosynthesis of Takeuchi Type III fractures provided equivalent biomechanical performance, restoring intact torsional stiffness in each rotational direction and torsional displacement in internal rotation. Based on our second hypothesis, Takeuchi Type II and III LHFs treated with screw or plate osteosynthesis showed comparable biomechanical performance with no significant differences in fracture site displacement and construct stiffness under axial and torsional loading within each fixation technique.

The integrity of the lateral hinge is crucial for maintaining the biomechanical stability of the MOWHTO [4, 12]. Besides smoking and obesity [11, 23], LHFs have been discussed as a risk factor leading to delayed union, non‐union, and loss of correction due to the increased micromovements across the osteotomy gap [30, 32]. However, the high incidence of LHFs compared to the relatively low rates of impaired bone healing (50% vs. 10%) raises the question which Takeuchi fracture type should be considered clinically relevant. To address this, Chen et al. [4] investigated the Takeuchi fracture type‐dependent axial stability of MOWHTO in a synthetic bone model. They have shown that Takeuchi Type II and III fractures resulted in a significantly greater medial osteotomy gap displacement of 0.5 mm under 800 N axial loading and a 10%–26% reduction in ultimate failure load compared to constructs with preserved lateral hinges. In contrast, Takeuchi Type I fractures did not affect the axial stability of the bone‐implant construct. Based on these findings, the authors concluded that Type III fractures were the most unstable and should be managed cautiously with delayed weight‐bearing and/or additional fixation.

In a recent finite element simulation, Chen et al. [5] investigated the mechanical properties in MOWHTO for different types of LHFs fixed with medial and bilateral locking plates. Under 2000 N axial compression in 0° knee flexion, plate fixation of the LHFs was able to restore the osteotomy gap shortening distance of 0.7 mm after Takeuchi type II and III fractures to the initial stability of 0.5 mm. These findings are similar to those of the present study, in which a 0.2 mm reduction in axial displacement was achieved with a plate fixation of the fractured lateral hinge, although lower axial compression loads (2000 N vs. 720 N in present study) were applied. However, Chen et al. did not consider the shear forces acting across the osteotomy that occur during normal gait when the knee joint is axially loaded in flexion [5, 31]. Therefore, the present study also investigated the effect of additional hinge fixation under torsional loading. In addition to restoring axial stability, plate fixation of Takeuchi Type II and III fractures also restored intact torsional hinge displacement of <0.2 mm.

The results of the present study are clinically relevant considering that impaired bone healing of the osteotomy gap, possibly caused by LHFs, remains a major concern in MOWHTO [24]. In previous in vivo studies of fracture healing, distraction and shear stress were found to inhibit bone healing, with a fracture gap >2 mm and a rotational displacement magnitude >0.2–1.0 mm being critical thresholds for impaired fracture healing [2, 13]. Consistent with these findings, Dorofeev et al. [8] reported significantly higher non‐union rates in patients with displaced LHFs (15.4% vs. 1.8%). In their retrospective analysis of 94 patients, the non‐union rate was particularly increased for LHFs with secondary dislocation >2 mm compared to undisplaced or primary displaced fractures >2 mm (25.0% vs. 6.8% and 1.8%), emphasizing that unstable LHFs lead to impaired bone healing of the osteotomy. In the current study, Takeuchi Type II and III fractures significantly increased torsional displacement across the osteotomy beyond this critical threshold of 2 mm in each torsional direction, suggesting that these fractures are prone to impaired bone healing and may require osteosynthetic treatment. Additional fixation of these fractures restored torsional displacement to the same level as that of the non‐hinge fractured bone. Thus, based on the current data, Takeuchi Type II fractures should be fixed with a plate, while Takeuchi Type III fractures should be addressed with either a plate or screw fixation to reduce the risk of non‐union or malunion.

The present study has several limitations. First, the biomechanical testing simulated forces acting at time zero when biological factors and osseous integration processes were not considered. Second, cadaveric knee specimens of older age (age 72.2 ± 7.0 years) were used, which might not necessarily reflect the bone quality of patients treated with MOWHTO. Nonetheless, proximal tibiae of all knees were assessed for BMD to ensure biomechanical testing in non‐osteoporotic specimens. Third, an axial load of 720 N and a torsional load of 10 Nm were applied in this study, which might not reflect the loading conditions during normal gait, when the knee bears up to four times the body weight [31]. Nonetheless, these loads were chosen to ensure consistency with the current literature [28] and to investigate hinge displacement at pre‐destructive loads to simulate early post‐operative rehabilitation. It should be noted, however, that higher axial loads may have altered the results. Therefore, the absolute values cannot be extrapolated 1:1 to the clinical situation in humans, although the conceptual findings are considered to be of great importance for the development of fracture‐specific treatment strategies for LHF. Fourth, the different fixation techniques were tested serially on the same specimen so that each fixation could affect the stability of the following fixation. Finally, the present study only examined displacement at the hinge site under axial compression and torsional loading. Other loading conditions that may also affect bone healing were not investigated [2, 13].

## CONCLUSION

LHFs after MOWHTO resulted in decreased bone‐implant construct stiffness and increased fracture site displacement. Additional plate fixation of Takeuchi Type II fractures was the construct with the highest stiffness, restoring the axial and torsional stability to a MOWHTO with an intact hinge. Screw and plate fixation of Takeuchi Type III fractures provided equivalent stability and restored the torsional and axial stability of the MOWHTO. In case of a Takeuchi Type II or III fracture, surgeons should consider additional plate or screw osteosynthesis of the fractured hinge to best restore the stability of the MOWHTO, which may potentially reduce the risk of loss of correction and impaired bone healing.

## AUTHOR CONTRIBUTIONS

**Christian Peez**: Conception and design, testing and data acquisition, statistical analysis and writing. **Adrian Deichsel**: Internal Review. **Ivan Zderic**: Testing and data acquisition, statistical analysis and internal review. **R. Geoff Richards**: Internal review. **Ludmil Drenchev**: Internal review. **Hristo K. Skulev**: Internal review. **Boyko Gueorguiev**: Internal review. **Michael Raschke**: Internal review. **Christoph Kittl**: Internal review. **Elmar Herbst**: Conception and design, writing and internal review.

## CONFLICT OF INTEREST STATEMENT

Elmar Herbst is Deputy Editor‐in‐Chief for the Knee Surgery, Sports Traumatology and Arthroscopy (KSSTA). The remaining authors declare no conflicts of interest.

## ETHICS STATEMENT

Institutional Review Board approval was obtained from the AO Research Institute Davos to conduct this biomechanical study (PP2115, 6 February 2018). The donors gave informed consent to the use of their corpses in medical science during their lifetime, so that the specimens were dissected and biomechanically tested in accordance with the relevant guidelines and regulations.

## Data Availability

Data are available from the corresponding author upon reasonable request.

## References

[1] Ackermann, J. , Merkely, G. , Arango, D. , Mestriner, A.B. & Gomoll, A.H. (2020) The effect of mechanical leg alignment on cartilage restoration with and without concomitant high tibial osteotomy. Arthroscopy: The Journal of Arthroscopic & Related Surgery, 36, 2204–2214. Available from: 10.1016/j.arthro.2020.04.019 32353621

[2] Barcik, J. & Epari, D.R. (2021) Can optimizing the mechanical environment deliver a clinically significant reduction in fracture healing time? Biomedicines, 9, 691. Available from: 10.3390/biomedicines9060691 34207370 PMC8234230

[3] Chahla, J. , Murray, I.R. , Robinson, J. , Lagae, K. , Margheritini, F. , Fritsch, B. et al. (2019) Posterolateral corner of the knee: an expert consensus statement on diagnosis, classification, treatment, and rehabilitation. Knee Surgery, Sports Traumatology, Arthroscopy, 27, 2520–2529. Available from: 10.1007/s00167-018-5260-4 30478468

[4] Chen, P. , Zhan, Y. , Zhan, S. , Li, R. , Luo, C. & Xie, X. (2021) Biomechanical evaluation of different types of lateral hinge fractures in medial opening wedge high tibial osteotomy. Clinical Biomechanics, 83, 105295. Available from: 10.1016/j.clinbiomech.2021.105295 33662653

[5] Chen, Y.N. , Chuang, C.H. , Yang, T.H. , Chang, C.W. , Li, C.T. , Chang, C.J. et al. (2020) Computational comparison of different plating strategies in medial open‐wedge high tibial osteotomy with lateral hinge fractures. Journal of Orthopaedic Surgery and Research, 15, 409. Available from: 10.1186/s13018-020-01922-0 32928260 PMC7489014

[6] Constantin, H. , Salmon, L.J. , Russell, V. , Sundaraj, K. , Roe, J.P. & Pinczewski, L.A. (2024) 20‐year Outcomes of high tibial osteotomy: determinants of survival and functional outcome. The American Journal of Sports Medicine, 52, 344–351. Available from: 10.1177/03635465231217742 38243788

[7] Dawson, M.J. , Ollivier, M. , Menetrey, J. & Beaufils, P. (2023) Osteotomy around the painful degenerative varus knee: a 2022 ESSKA formal consensus. Knee Surgery, Sports Traumatology, Arthroscopy, 31, 3041–3043. Available from: 10.1007/s00167-022-07024-0 35697873

[8] Dorofeev, A. , Tylla, A. , Benco, M. , Drescher, W. & Stangl, R. (2020) Opposite hinge fractures in high tibial osteotomy: a displacement subtype is more critical than a fracture type. European Journal of Orthopaedic Surgery & Traumatology, 30, 297–305. Available from: 10.1007/s00590-019-02549-6 31506790

[9] Faul, F. , Erdfelder, E. , Buchner, A. & Lang, A.‐G. (2009) Statistical power analyses using G*Power 3.1: tests for correlation and regression analyses. Behavior Research Methods, 41, 1149–1160. Available from: 10.3758/BRM.41.4.1149 19897823

[10] Ferner, F. , Lutter, C. & Harrer, J. (2024) Handlungsalgorithmus: vorgehen Bei Hinge‐Fraktur bei Tibiakopfosteotomie. Knie Journal, 6, 240–242. Available from: 10.1007/s43205-024-00288-1

[11] Floerkemeier, S. , Staubli, A.E. , Schroeter, S. , Goldhahn, S. & Lobenhoffer, P. (2014) Does obesity and nicotine abuse influence the outcome and complication rate after open‐wedge high tibial osteotomy? A retrospective evaluation of five hundred and thirty three patients. International Orthopaedics, 38, 55–60. Available from: 10.1007/s00264-013-2082-3 24022738 PMC3890126

[12] Franulic, N. , Muñoz, J.T. , Figueroa, F. , Innocenti, P. & Gaggero, N. (2023) Lateral hinge fracture in medial opening wedge high tibial osteotomy: a narrative review. EFORT Open Reviews, 8, 572–580. Available from: 10.1530/EOR-22-0103 37395709 PMC10321048

[13] Glatt, V. , Evans, C.H. & Tetsworth, K. (2016) A concert between biology and biomechanics: the influence of the mechanical environment on bone healing. Frontiers in Physiology, 7, 678. Available from: 10.3389/fphys.2016.00678 28174539 PMC5258734

[14] Gulagaci, F. , Jacquet, C. , Ehlinger, M. , Sharma, A. , Kley, K. , Wilson, A. et al. (2020) A protective hinge wire, intersecting the osteotomy plane, can reduce the occurrence of perioperative hinge fractures in medial opening wedge osteotomy. Knee Surgery, Sports Traumatology, Arthroscopy, 28, 3173–3182. Available from: 10.1007/s00167-019-05806-7 31773202

[15] Hantes, M.E. , Natsaridis, P. , Koutalos, A.A. , Ono, Y. , Doxariotis, N. & Malizos, K.N. (2018) Satisfactory functional and radiological outcomes can be expected in young patients under 45 years old after open wedge high tibial osteotomy in a long‐term follow‐up. Knee Surgery, Sports Traumatology, Arthroscopy, 26, 3199–3205. Available from: 10.1007/s00167-017-4816-z 29189881

[16] Kang, K.T. , Koh, Y.G. , Lee, J.A. , Lee, J.J. & Kwon, S.K. (2020) Biomechanical effect of a lateral hinge fracture for a medial opening wedge high tibial osteotomy: finite element study. Journal of Orthopaedic Surgery and Research, 15, 63. Available from: 10.1186/s13018-020-01597-7 32085786 PMC7035662

[17] Khakha, R.S. , Bin Abd Razak, H.R. , Kley, K. , van Heerwaarden, R. & Wilson, A.J. (2021) Role of high tibial osteotomy in medial compartment osteoarthritis of the knee: Indications, surgical technique and outcomes. Journal of Clinical Orthopaedics and Trauma, 23, 101618. Available from:10.1016/j.jcot.2021.101618 35070682 PMC8758909

[18] Kumagai, K. , Yamada, S. , Nejima, S. , Muramatsu, S. , Akamatsu, Y. & Inaba, Y. (2020) Lateral hinge fracture delays healing of the osteotomy gap in opening wedge high tibial osteotomy with a beta‐tricalcium phosphate block. The Knee, 27, 192–197. Available from: 10.1016/j.knee.2019.10.027 31883759

[19] Lau, L.C.M. , Fan, J.C.H. , Chung, K.Y. , Cheung, K.W. , Man, G.C.W. , Hung, Y.W. et al. (2021) Satisfactory long‐term survival, functional and radiological outcomes of open‐wedge high tibial osteotomy for managing knee osteoarthritis: minimum 10‐year follow‐up study. Journal of Orthopaedic Translation, 26, 60–66. Available from: 10.1016/j.jot.2020.03.003 33437624 PMC7773956

[20] Lee, B.S. , Jo, B.K. , Bin, S.I. , Kim, J.M. , Lee, C.R. & Kwon, Y.H. (2019) Hinge Fractures are underestimated on plain radiographs after open wedge proximal tibial osteotomy: evaluation by computed tomography. The American Journal of Sports Medicine, 47, 1370–1375. Available from: 10.1177/0363546519836949 30986094

[21] Lee, O.S. & Lee, Y.S. (2018) Diagnostic value of computed tomography and risk factors for lateral hinge fracture in the open wedge high tibial osteotomy. Arthroscopy: The Journal of Arthroscopic & Related Surgery, 34, 1032–1043. Available from: 10.1016/j.arthro.2017.08.310 29229417

[22] Lee, S.S. , Celik, H. & Lee, D.H. (2018) Predictive factors for and detection of lateral hinge fractures following open wedge high tibial osteotomy: plain radiography versus computed tomography. Arthroscopy: The Journal of Arthroscopic & Related Surgery, 34, 3073–3079. Available from: 10.1016/j.arthro.2018.06.041 30292595

[23] Meidinger, G. , Imhoff, A.B. , Paul, J. , Kirchhoff, C. , Sauerschnig, M. & Hinterwimmer, S. (2011) May smokers and overweight patients be treated with a medial open‐wedge HTO? Risk factors for non‐union. Knee Surgery, Sports Traumatology, Arthroscopy, 19, 333–339. Available from: 10.1007/s00167-010-1335-6 21153542

[24] Miltenberg, B. , Puzzitiello, R.N. , Ruelos, V.C.B. , Masood, R. , Pagani, N.R. , Moverman, M.A. et al. (2024) Incidence of complications and revision surgery after high tibial osteotomy: a systematic review. The American Journal of Sports Medicine, 52, 258–268. Available from: 10.1177/03635465221142868 36779579

[25] Nakamura, R. , Komatsu, N. , Fujita, K. , Kuroda, K. , Takahashi, M. , Omi, R. et al. (2017) Appropriate hinge position for prevention of unstable lateral hinge fracture in open wedge high tibial osteotomy. The Bone & Joint Journal, 99–b, 1313–1318. Available from: 10.1302/0301-620X.99B10.BJJ-2017-0103.R1 28963152

[26] Nakamura, R. , Komatsu, N. , Murao, T. , Okamoto, Y. , Nakamura, S. , Fujita, K. et al. (2015) The validity of the classification for lateral hinge fractures in open wedge high tibial osteotomy. The Bone & Joint Journal, 97–b, 1226–1231. Available from: 10.1302/0301-620X.97B9.34949 26330589

[27] Palmer, J. , Getgood, A. , Lobenhoffer, P. , Nakamura, R. & Monk, P. (2024) Medial opening wedge high tibial osteotomy for the treatment of medial unicompartmental knee osteoarthritis: a state‐of‐the‐art review. Journal of ISAKOS, 9, 39–52. Available from: 10.1016/j.jisako.2023.10.004 37839705

[28] Peez, C. , Grosse‐Allermann, A. , Deichsel, A. , Raschke, M.J. , Glasbrenner, J. , Briese, T. et al. (2023) Additional plate fixation of hinge fractures after varisation distal femoral osteotomies provides favorable torsional stability: a biomechanical study. The American Journal of Sports Medicine, 51, 3732–3741. Available from: 10.1177/03635465231206947 37936394 PMC10691291

[29] Schroeder, S. , Jaeger, S. , Schwer, J. , Seitz, A.M. , Hamann, I. & Werner, M. et al. (2022) Accuracy measurement of different marker based motion analysis systems for biomechanical applications: a round robin study. PLoS One, 17, e0271349. Available from: 10.1371/journal.pone.0271349 35816503 PMC9273086

[30] Schröter, S. , Freude, T. , Kopp, M.M. , Konstantinidis, L. , Döbele, S. , Stöckle, U. et al. (2015) Smoking and unstable hinge fractures cause delayed gap filling irrespective of early weight bearing after open wedge osteotomy. Arthroscopy: The Journal of Arthroscopic & Related Surgery, 31, 254–265. Available from: 10.1016/j.arthro.2014.08.028 25442655

[31] Shelburne, K.B. , Torry, M.R. & Pandy, M.G. (2005) Muscle, ligament, and joint‐contact forces at the knee during walking. Medicine & Science in Sports & Exercise, 37, 1948–1956. Available from: 10.1249/01.mss.0000180404.86078.ff 16286866

[32] Takeuchi, R. , Ishikawa, H. , Kumagai, K. , Yamaguchi, Y. , Chiba, N. , Akamatsu, Y. et al. (2012) Fractures around the lateral cortical hinge after a medial opening‐wedge high tibial osteotomy: a new classification of lateral hinge fracture. Arthroscopy: The Journal of Arthroscopic & Related Surgery, 28, 85–94. Available from: 10.1016/j.arthro.2011.06.034 21982387

[33] Zaffagnini, S. , Romandini, I. , Filardo, G. , Dal Fabbro, G. & Grassi, A. (2023) Meniscal allograft transplantation, anterior cruciate ligament reconstruction, and valgus high tibial osteotomy for meniscal‐deficient, unstable, and varus knees: surgical technique and clinical outcomes. International Orthopaedics, 47, 2523–2535. Available from: 10.1007/s00264-023-05846-2 37316682

